# Cerebral blood flow imbalance is associated with motor outcome after pediatric arterial ischemic stroke

**DOI:** 10.1371/journal.pone.0223584

**Published:** 2019-10-11

**Authors:** Rebekka Leistner, Regula Everts, Andrea Federspiel, Salome Kornfeld, Nedelina Slavova, Leonie Steiner, Roland Wiest, Maja Steinlin, Sebastian Grunt

**Affiliations:** 1 Division of Neuropediatrics, Development and Rehabilitation, University Children’s Hospital, Inselspital, Bern University Hospital, University of Bern, Bern, Switzerland; 2 Department of Diabetes, Endocrinology, Clinical Nutrition and Metabolism, Inselspital, Bern University Hospital, University of Bern, Bern, Switzerland; 3 Psychiatric Neuroimaging Unit, Translational Research Center, University Hospital of Psychiatry, University of Bern, Bern, Switzerland; 4 Department of Diagnostic and Interventional Neuroradiology, Inselspital, Bern University Hospital, University of Bern, Bern, Switzerland; Nuovo Ospedale Prato (NOP) Santo Stefano, USL Toscana Centro, ITALY

## Abstract

Cerebral hemodynamics after arterial ischemic stroke (AIS) in children are largely unknown. This study aims to explore long-term cerebral perfusion balance of vital tissue and its relation to motor outcome after childhood AIS. Patients diagnosed with childhood AIS (≤16 years at diagnosis, time since stroke ≥2 years) and typically developing peers were examined. Hemiparesis was classified according to the Pediatric Stroke Outcome Measure. Manual ability was assessed using the ABILHAND-Kids questionnaire. Cerebral blood flow was measured by arterial spin labeling and analyzed in the following brain regions: the hemispheres, the territory of the anterior cerebral artery (ACA), the middle cerebral artery (MCA), and in subregions of the MCA territory (MCA anterior, middle, posterior). To assess cerebral perfusion balance, laterality indices were calculated using cerebral blood flow in the ipsi- and contralesional hemisphere. Laterality indices were compared between stroke patients with and without hemiparesis, and peers. Twenty participants diagnosed with AIS were included (12 boys, 8 girls; mean age 14.46±4.96 years; time since stroke 8.08±3.62 years); 9 (45%) were diagnosed with hemiparesis. Additionally, 47 typically developing peers (21 boys, 26 girls; mean age 14.24±5.42 years) were studied. Laterality indices were higher in stroke patients and oriented to the contralesional hemisphere in all brain regions except the ACA territory and MCA posterior subregion. This was significantly different from peers, who showed balanced laterality indices. There was a significant correlation between laterality indices and manual ability, except in the ACA territory. AIS is associated with long-term alterations of cerebral blood flow in vital tissue, even in patients without hemiparesis. The degree of imbalance of cerebral perfusion in children after AIS is associated with manual ability.

## Introduction

Pediatric arterial ischemic stroke (AIS) is rare. Its incidence is 1.2 to 7.9 cases per 100,000 person-years and it occurs most frequently in children of pre-school age and in boys [1–3]. Neonatal AIS is diagnosed in 5 to 43 cases per 100,000 live births per year [4–6]. Both childhood and neonatal AIS can be associated with considerable long-term morbidity, such as epilepsy, cognitive disturbance, behavioral problems, and neuro-motor impairment [7]. Hemiparesis is an important sequela in children and neonates diagnosed with AIS [3,6,7]. It can lead to decreased manual ability and thus to reduced health-related quality of life [8]. The trajectory of motor recovery after AIS during childhood differs according to the child’s age at stroke onset [9].

Understanding the neuroplastic processes after childhood AIS can improve management and help target rehabilitation strategies. Therefore, it is important to study cerebral, post-ischemic changes in detail. Diffusion tensor imaging, functional magnetic resonance imaging (fMRI) and, more recently, resting state fMRI [10], have led to a better understanding of the structural and functional changes in the child’s brain after ischemia. A relatively new approach is arterial spin labeling (ASL), a non-invasive MRI technique that allows cerebral blood flow (CBF) in the brain to be quantified. As CBF mirrors the brain’s metabolic demands and neuronal activity, the measurement of CBF in a stroke-affected region provides information about its activity and functional recovery [11]. ASL has been used to measure CBF changes in adults during the acute and chronic phase of AIS. A longitudinal study showed that sustained perfusion imbalance within the sensorimotor network was associated with poor motor recovery in the hand. The authors concluded that the degree of interhemispheric balance may be crucial for motor recovery after AIS [12]. One of the few pediatric studies examined 10 children during the acute phase of AIS by measuring CBF in the region of infarct. Hypoperfused lesions were found to be associated with larger acute and follow-up stroke volumes than normal perfusion or hyperperfusion [11]. CBF may be altered not only in the acute, but also in the chronic phase of AIS. Hypoperfusion may persist in regions that are anatomically, but not functionally intact. The reduced CBF seems to be sufficient to ensure viability of these regions, but not to allow full functional recovery [13,14,15].

While previous studies mainly focused on adults, few investigations have looked at the long-term characteristics of cerebral hemodynamics in children after AIS and its relation to motor outcome. The present study aimed to examine long-term cerebral perfusion balance of vital tissue and its relation to motor outcome in patients diagnosed with childhood or neonatal AIS ≥2 years ago (i.e. chronic phase of AIS). A further aim was to assess the relationship between CBF and age and sex in stroke patients and peers. The study was based on the following hypotheses: A. CBF in vital tissue is less balanced in stroke patients than in peers; B. CBF in vital tissue is less balanced in stroke patients with hemiparesis than in stroke patients without hemiparesis; C. Better balanced cerebral perfusion after pediatric AIS is related to better manual ability; and D. The relationship between CBF and age is similar in stroke patients and peers.

## Material and methods

### Participants

Participants were recruited from 2014 through 2016 as a part of the Hemispheric Reorganisation Study (HERO) [16], which was approved by the Cantonal Ethics Committee of Bern, Switzerland, and performed according to the Code of Ethics of the World Medical Association (Declaration of Helsinki). Written informed consent for participation was given by the participants themselves (≥18 years of age) or by a parent or guardian. Patients diagnosed with AIS were identified by the Swiss Neuropaediatric Stroke Registry (SNPSR)–a multicenter, prospective, and population-based registry including children diagnosed with AIS aged ≤16 years [3]. Patients diagnosed with AIS at least 2 years before recruitment, confirmed by MRI and/or computed tomography, were considered for inclusion. A convenience sample of typically developing peers was recruited from schools and personal contacts. Exclusion criteria for both patients and peers were: ferrous implants, behavioral problems or claustrophobia (making MRI impossible). Patients with bilateral lesions were also excluded. Clinical examination, MRI and ASL scans were all performed on the same day at the Inselspital, Bern University Hospital, and University of Bern, Switzerland. Information about etiology and treatment in the acute phase of stroke was collected from the SNPSR and etiology was classified according to the CASCADE criteria [17].

### Clinical examination

A standardized neurological examination was performed on all participants by a research physician, which included the following:

The Pediatric Stroke Outcome Measure (PSOM) was performed to assess disease-specific outcome. The PSOM assesses neurological deficits after childhood stroke and consists of 5 subscales (right and left sensorimotor, language production, language comprehension, and cognitive/behavior) [18]. The sensorimotor subscale of the PSOM was used to classify the presence or absence of hemiparesis (0 = no deficit, 0.5 = mild deficit with normal function, 1 = moderate deficit with decreased function, 2 = severe deficit with missing function). Patients with scores ≥0.5 were classified as hemiparetic.

The Edinburgh Handedness Inventory (EHI) [19] was used to assess hand dominance. This questionnaire evaluates the hand preference during 10 everyday life activities and allows a person’s handedness to be assigned a laterality quotient, ranging from −100 (using the left hand only) to 100 (using the right hand only).

Manual ability was assessed with the ABILHAND-Kids questionnaire [20]. This is a unidimensional measure for 21 mostly bimanual activities, which are rated as impossible (0), difficult (1), or easy (2) to perform. The score was transformed into the ABILHAND-Kids measure ranging from +6.68 (all items easy to perform) to −6.75 (all items impossible to perform).

### Neuroimaging

The MRI protocol was adopted from the HERO study, in which supplementary sequences were performed in addition to the ASL. In order to compare CBF values within and between subjects, it was mandatory to follow the study protocol (which includes the exact settings of the MR sequences). MRI was performed on a 3T whole-body MRI system (Magnetom Prisma, Siemens Medical Systems, Erlangen, Germany) with a 64-channel radiofrequency head coil.

#### Structural imaging

A high-resolution 3D T1-weighted magnetization-prepared rapid-acquisition gradient-echo (MP-RAGE) was recorded for every subject with the following parameters: echo time (TE) = 2.96ms; repetition time (TR) = 2530ms; matrix size, 256×256; field of view, 256mm^2^; 160 slices; and slice thickness, 1mm with isovoxel resolution of 1×1×1mm. The scan duration was 5min 05s.

Topographical lesion location was based on original medical reports, the SNPSR database, and the acquired anatomical MRI images. Lesions were classified according to their anatomical location (cortical, subcortical, or both cortical and subcortical) and the hemisphere(s) involved (left or right) by a board-certified neuroradiologist (N.S.) with 10 years of experience. Ischemic lesions were manually traced as a region of interest to create lesion masks. For further analyses, stroke volume was used relative to the total brain volume, calculated as stroke volume [cm^3^]/total brain volume [cm^3^].

#### Arterial spin labeling

CBF was assessed using a pseudo-continuous arterial spin labeling (pCASL) sequence [21,22]. An alternating sequence of label and control images was acquired. Labeling was performed at 80mm below the isocenter of the imaging region and a post-labeling delay (PLD) of 1.25s was set with a label time of 1.6s. Since the present study was conducted before the publication of the latest recommended values [23], PLD was not set to 1.5s as now recommended. Using the statistical parametric mapping (SPM12, Wellcome Department of Imaging Neuroscience, London, England [24]) “segmentation” option, the gray matter (GM) mask can be computed as a separate map by choosing the segmented GM tissue map, which is then binarized using a threshold value of GM intensity of 0.7. Both the segmented images are in normalized standard space, and the M0 images as well as the CBF maps are in the normalized standard space. In summary, all MRI modalities were processed so that a normalized standard space was available to ensure the extraction of CBF values for homologous brain regions.

Images were acquired using the following parameters: TE = 12ms; TR = 3400ms; field of view, 230mm^2^; matrix size, 64×64; flip angle 90°; voxel size, 3.6×3.6×6.0mm. A total of 16 slices with a slice thickness of 6mm were recorded sequentially from inferior to superior. Each pCASL measurement was repeated 120 times. Additionally, one M0 image for tissue at equilibrium magnetization was recorded with TR = 8000ms and PLD = 5000ms. All other parameters were unchanged. The duration of the ASL scan was 6min 58s.

For the analysis of the ASL data, we used SPM12 and MATLAB (MathWorks Inc.; version R2017a). First, we realigned all ASL time series to correct for motion artifacts. Then, each subject’s anatomical T1 image was segmented into GM, white matter, and cerebrospinal fluid (CSF). The estimation of CBF can be performed with the ASL technique. In fact, a calibrated CBF measure can be obtained by one-compartment models [25,26] solving the following equation:

$$CBF=\left(\frac{\lambda \cdot \Delta M}{2\cdot \alpha \cdot M_{0}\cdot T_{1b}}\right)\cdot \left(\frac{1}{e^{-w/T_{1b}}-e^{-\left(\tau +w\right)/T_{1b})}}\right)$$

The variables are as follows: post-labeling delay (ω) (PLD), labeling duration (τ), blood/tissue water partition coefficient λ = 0.9g/mL, and labeling efficiency α = 0.85 [21]. In the human brain, and for 3.0T, the decay time for labeled blood T1b = 1650ms is assumed. Moreover, M_0_ are the equilibrium brain tissue magnetization images [25,27,28] and were acquired in separate runs. ΔΜ represents the time series obtained by subtraction of control and label images.

Each CBF map was then masked with the segmented GM anatomical images. We used a threshold of 0.7 for the creation of each GM-mask that was then applied to each CBF map. To exclude ischemic lesions from CBF measurements, the lesion masks generated from the T1-weighted anatomical images were superimposed on the CBF map. Thus, CBF was only measured in anatomically intact tissue. Resulting mean CBF maps were then co-registered to the anatomical scans, normalized to the Montreal Neurological Institute coordinate system, and spatially smoothed with a Gaussian kernel (8mm, full-width at half-maximum). Mean MCA and MCA middle CBF maps (see below for the definitions of these brain areas) were left in native space, because normalization of specific brain regions is difficult in children.

**Fig 1:**
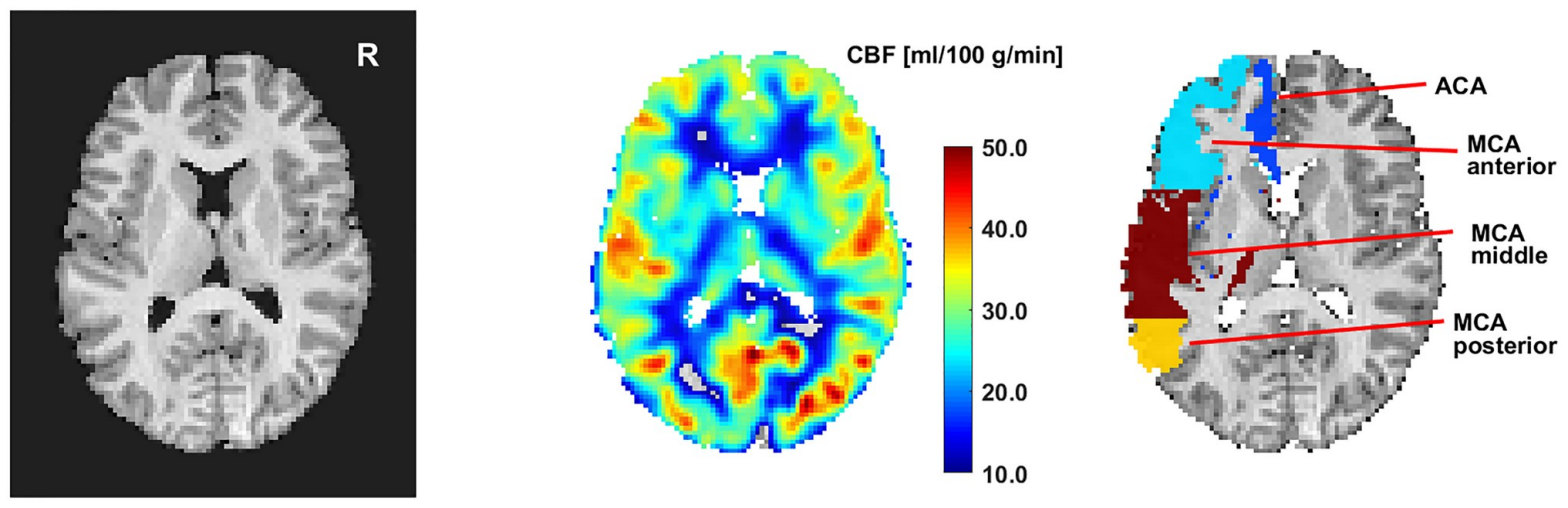
MRI, ASL perfusion map and analyzed brain regions. The left panel shows an axial brain MRI, the middle panel an ASL perfusion map with colors indicating the regional cerebral perfusion in ml/100g/min. The right panel shows the analyzed vascular territories of the anterior cerebral artery (ACA), middle cerebral artery (MCA) and subregions (MCA anterior, middle, posterior).

CBF was measured throughout the brain and separately in each of the hemispheres. In addition, CBF in the territories of the ACA and MCA was measured. Furthermore, the MCA territory was divided into 3 subregions (MCA anterior, middle, and posterior) in which CBF was analyzed separately (see Fig 1). These regions were chosen because we were specifically interested in motor function and the different components of the motor network are located in these areas.

#### CBF laterality indices

The degree of CBF lateralization was assessed by calculating CBF laterality indices (LIs). LIs were calculated according to the following formula based upon Wiest et al. [12]:

$$CBF\;LI=\frac{CBFc-CBFi}{CBFc+CBFi}$$

c and i indicate the contra- and ipsilesional hemisphere. In peers, dominant and non-dominant hemisphere, as determined by the Edinburgh Handedness Inventory, were used instead. A positive LI signifies a CBF lateralization to the contralesional hemisphere (in peers to the dominant hemisphere) and a negative LI signifies a CBF lateralization to the ipsilesional hemisphere (in peers to the non-dominant hemisphere). LIs were calculated for hCBF, MCA CBF and MCA middle CBF.

To control for subject motion leading to imprecise CBF measurements, deviations from the initial position were monitored during the ASL scan. Deviations were measured along the x-, y- and z-axes in mm (x, y, z) and in radians (α, β, γ).

### Statistical analysis

Statistical analysis was performed with IBM SPSS 23.0 [29]. Data was tested for normality with the Shapiro-Wilk test. To compare mean values between two groups, independent samples t-tests (normally distributed variables) or Mann-Whitney U-tests (non-normally distributed variables) were used. To correct for different age distributions among the groups, which could influence global CBF, group comparisons were performed by analyses of covariance with age at examination as a covariate. Comparisons of mean values between more than two groups were performed by univariate analyses of variance (normally distributed variables) or Kruskal-Wallis tests (non-normally distributed variables). For post-hoc pairwise comparison Dunn-Bonferroni tests were used. For correlation analyses, Pearson (normally distributed variables) or Spearman correlations (non-normally distributed variables) were applied. A *p*-value of <0.05 was considered statistically significant.

## Results

### Participants and clinical characteristics

Twenty-nine patients diagnosed with chronic AIS and 50 typically developing peers were recruited. Nine AIS patients were excluded for the following reasons: developmental delay or behavioral problems interfering with compliance (n = 2), bilateral lesions (n = 4), retainer artifacts (n = 1), error in T1-weighted anatomical image or ASL sequences (n = 2). Three peers were excluded due to error in ASL sequence (n = 2) or retainer artifacts (n = 1).

The final study population consisted of 20 AIS patients and 47 peers. Nine patients (45%) were diagnosed with hemiparesis (PSOM 0.5: n = 3, PSOM 1: n = 4, PSOM 2: n = 2). Clinical characteristics are provided in Table 1; for detailed patient data please see S1 Table.

**Table 1:** Clinical characteristics in stroke patients with and without hemiparesis and peers.

	Peers	Stroke without hemiparesis	Stroke with hemiparesis	*p*-Value
Sex (m/f)	21/26	7/4	5/4	NA
Age at examination (y)	14.24 (5.42)	14.42 (4.77)	14.51 (5.48)	0.987†
Global CBF (ml/100g/min)	53.57 (12.16)	50.09 (14.52)	52.20 (10.43)	0.636‡
ABILHAND-Kids	6.47 (0.62)	5.35 (1.25)	3.06 (2.35)	<0.001*§
Age at stroke (y)	NA	7.33 (5.77)	4.47 (3.85)	0.220| |
Time since stroke (y)	NA	7.04 (3.89)	9.35 (2.99)	0.160#
Stroke volume	NA	0.68 (1.46)	2.84 (4.10)	0.095**

All values are given as mean (standard deviation).

*p<0.05

^†^ Univariate analysis of variance (F(2,64) = 0.01)

^‡^ Kruskal-Wallis test (χ2(2) = 0.91)

^§^ Kruskal-Wallis test (χ2(2) = 32.88)

^| |^ Independent samples t-test (t(18) = 1.27)

^#^ Independent samples t-test (t(18) = −1.46)

** Independent samples Mann-Whitney U-test (U = 72.00)

Stroke patients showed significantly lower ABILHAND-Kids measures than typically developing peers (stroke without hemiparesis vs peers: *p* = 0.002, stroke with hemiparesis vs peers: *p*<0.001). ABILHAND-Kids measures did not differ between patients with hemiparesis and patients without hemiparesis (see Table 1).

### Relation between global CBF, age at examination and sex in patients and typically developing peers

**Fig 2:**
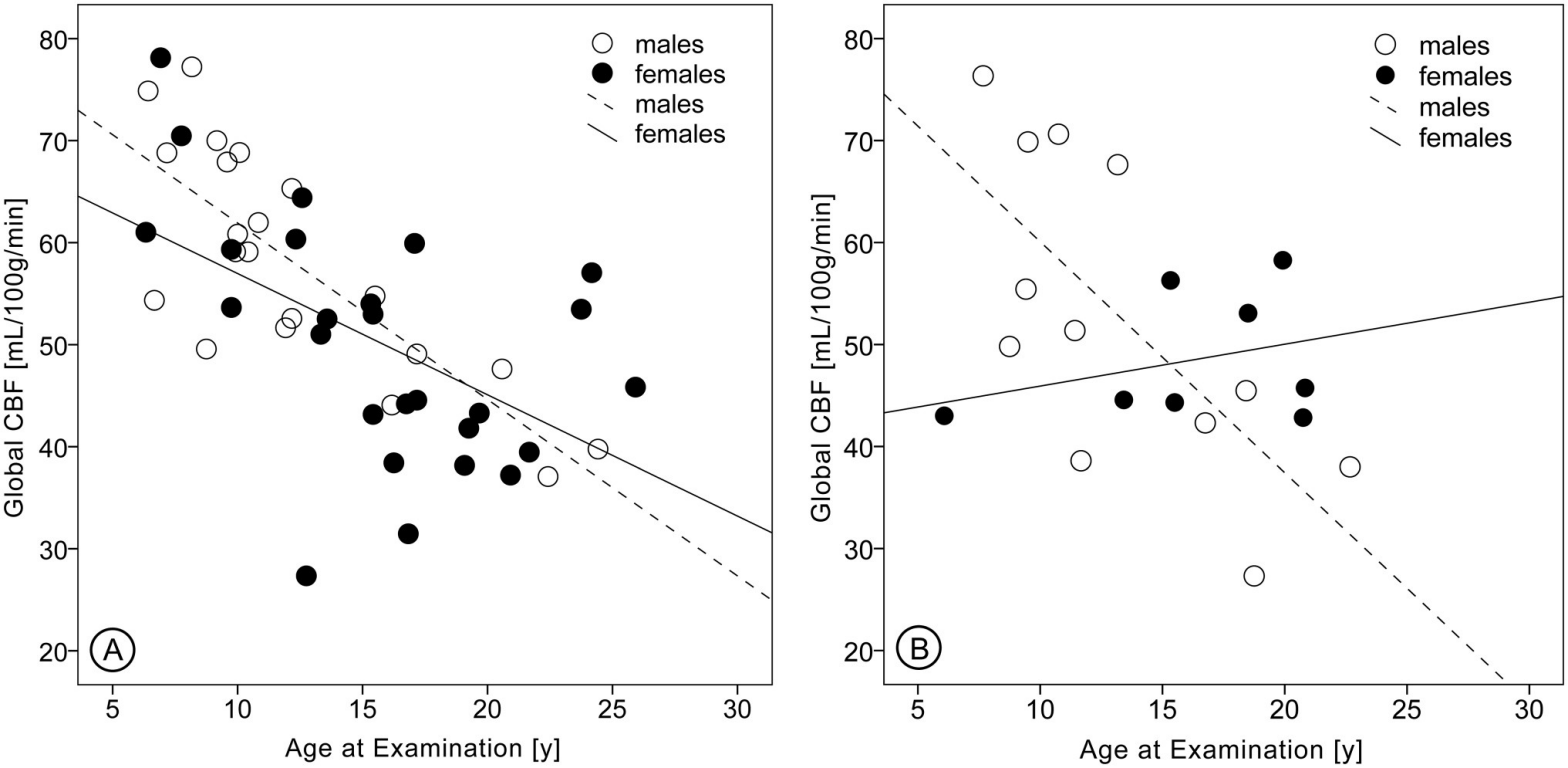
Global cerebral blood flow in peers and stroke patients. (A) The course of global cerebral blood flow (CBF) with increasing age in male and female peers and (B) in male and female stroke patients. Data points indicate participants; lines are regression curves best fitting the data points.

In both patients and peers a significant negative correlation between global CBF and age at examination was observed (r = −0.47, *p* = 0.036 and r = −0.67, *p*<0.001). In male and female peers as well as in male patients, global CBF decreased with increasing age. Global CBF in female patients, by contrast, increased with increasing age (Fig 2).

Significant differences in global CBF were observed between male and female peers (*p* = 0.040, mean (standard deviation) male: 57.83 (11.24) mL/100g/min, female: 50.13 (11.97) mL/100g/min). When corrected for different age distributions no difference was observed.

### Laterality indices in patients with and without hemiparesis and typically developing peers

**Fig 3:**
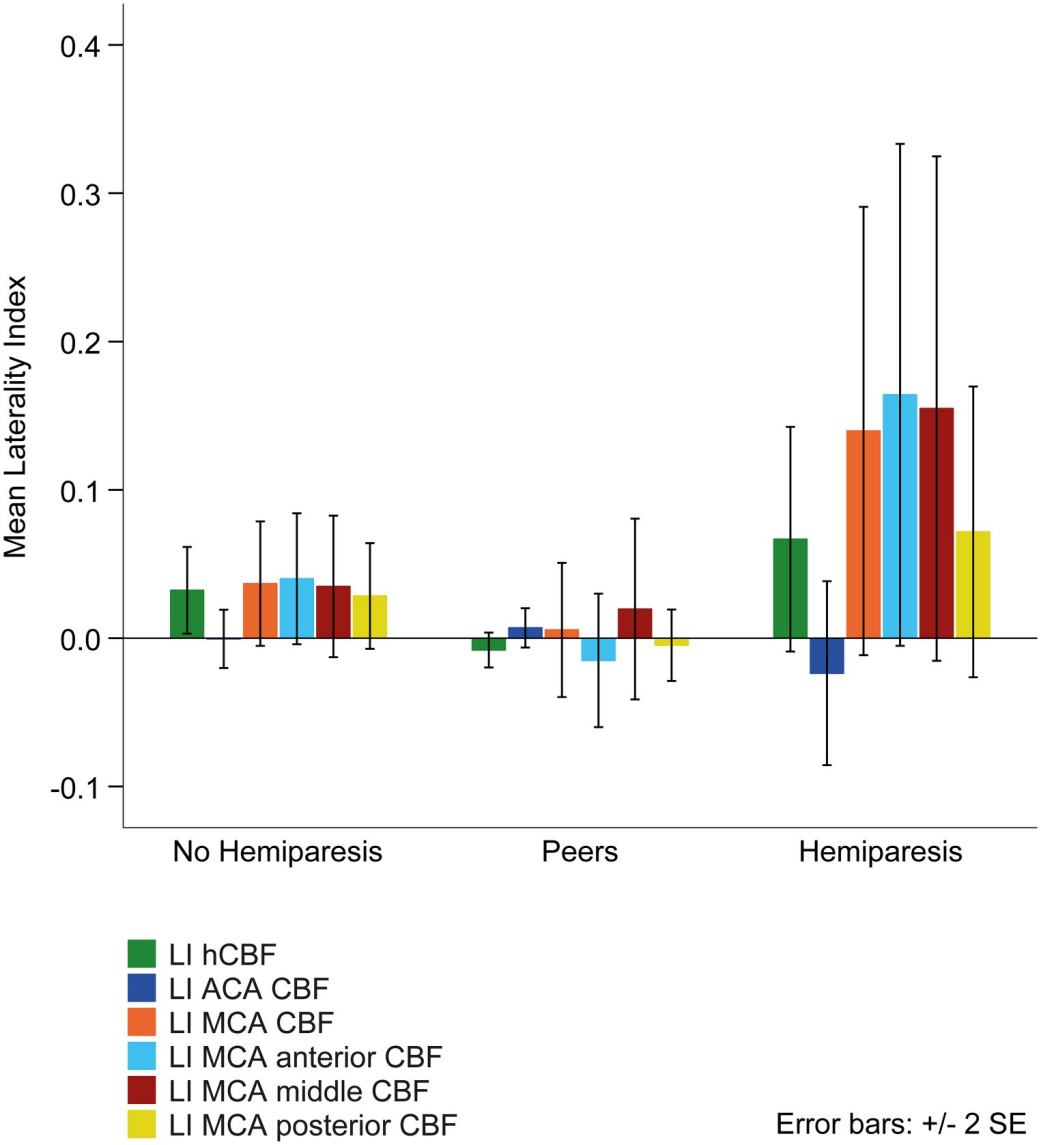
Cerebral blood flow laterality indices in patients with and without hemiparesis and in peers. Positive values indicate CBF lateralization to the contralesional hemisphere (and, in peers, to the dominant hemisphere); negative values indicate lateralization to the ipsilesional hemisphere (and, in peers, to the non-dominant hemisphere). Note the positive LIs in patients with and without hemiparesis (except LI ACA CBF), whereas LIs are near 0 in typically developing peers.

Differences in LIs between patients with and without hemiparesis and peers are presented in Table 2 and Fig 3.

**Table 2:** Mean laterality indices (LIs) and differences between typically developing peers and patients.

	LI hCBF	LI ACA	LI MCA CBF	LI MCA anterior CBF	LI MCA middle CBF	LI MCA posterior CBF
Group	Mean (standard deviation)					
No hemiparesis	0.032 (0.048)	0.0004 (0.033)	0.037 (0.070)	0.040 (0.073)	0.035 (0.079)	0.028 (0.059)
Hemiparesis	0.067 (0.114)	−0.024 (0.093)	0.140 (0.227)	0.164 (0.254)	0.155 (0.255)	0.072 (0.147)
Peers	−0.008 (0.040)	0.007 (0.045)	0.006 (0.155)	−0.015 (0.154)	0.020 (0.209)	−0.005 (0.083)
Compared groups	*p*-value					
All groups	<0.001*†	0.959‡	<0.001*§	0.020*| |	0.001*#	0.230**
No hemiparesis–Peers	0.001*	NA	0.015*	0.377	0.035*	NA
No hemiparesis–Hemiparesis	1.000	NA	1.000	1.000	1.000	NA
Hemiparesis–peers	0.004*	NA	0.001*	0.031*	0.003*	NA

* p<0.05.

^†^ Kruskal-Wallis test (χ2(2) = 19.60)

^‡^ Kruskal-Wallis test (χ2(2) = 0.084)

^§^ Kruskal-Wallis test (χ2(2) = 18.50)

^| |^ Kruskal-Wallis test (χ2(2) = 7.80)

^#^ Kruskal-Wallis test (χ2(2) = 14.70)

** Kruskal-Wallis test (χ2(2) = 2.94).

Pairwise group comparisons by Dunn-Bonferroni test. Significance values have been adjusted by the Bonferroni correction for multiple tests.

### Relationship between laterality indices, ABILHAND-Kids measures, stroke volume, age at stroke, and time since stroke

For correlations between LIs and these parameters see Table 3.

**Table 3:** Correlations (correlation coefficient r and *p* values).

	Stroke volume	Age at stroke	Time since stroke	ABIL-HAND-Kids	LI hCBF	LI ACA CBF	LI MCA CBF	LI MCA anterior CBF	LI MCA middle CBF	LI MCA posterior CBF
Stroke volume		-0.15	0.35	-0.65	0.26	-0.36	0.34	0.41	0.32	0.32
		0.532	0.126	0.002*	0.262	0.120	0.143	0.072	0.173	0.169
Age at stroke	−0.15		−0.44†	0.39	0.12	−0.20	0.02	−0.06	0.05	0.03
	0.532		0.053	0.090	0.617	0.389	0.922	0.791	0.847	0.889
Time since stroke	0.35	−0.44†		−0.12	−0.12	−0.10	0.20	−0.04	0.21	0.17
	0.126	0.053		0.625	0.605	0.691	0.394	0.870	0.387	0.462
ABILHAND-Kids	−0.65	0.39	−0.12		−0.41	0.004	−0.34	−0.32	−0.27	−0.25
	.002*	0.090	0.625		0.001*	0.977	0.005*	0.009*	0.030*	0.039*
LI hCBF	0.26	0.12	−0.12	−0.41		0.27	−0.90	−0.15	0.82	−0.03
	0.262	0.617	0.605	0.001*		0.027*	<0.001*	0.230	<0.001*	0.826
LI ACA CBF	−0.36	−0.20	−0.10	0.004	0.27		0.28	0.54	0.23	−0.45
	0.120	0.389	0.691	0.977	0.027*		0.021*	<0.001*	0.067	<0.001*
LI MCA CBF	0.34	0.02	0.20	−0.34	0.90	0.28		−0.18	0.94	−0.03
	0.143	0.922	0.394	0.005*	<0.001*	0.021*		0.148	<0.001*	0.815
LI MCA anterior CBF	0.41	0.06	−0.04	−0.32	−0.15	−0.54	−0.18		−0.19	0.50
	0.072	0.791	0.870	0.009*	0.230	<0.001*	0.148		0.131	<0.001*
LI MCA middle CBF	0.32	0.05	0.21	−0.27	0.82	0.23	0.94	−0.19		0.02
	0.173	0.847	0.387	0.030*	<0.001*	0.067	<0.001*	0.131		0.905
LI MCA posterior CBF	0.32	0.03	0.17	−0.25	−0.03	−0.45	−0.03	0.50	0.02	
	0.169	0.889	0.462	0.039*	0.826	<0.001*	0.815	<0.001*	0.905	

* p<0.05, unless otherwise noted: Spearman correlation.

^†^ Pearson correlation

## Discussion

This cross-sectional study examined cerebral hemodynamics in patients diagnosed with childhood AIS and in peers, using pCASL. To assess cerebral perfusion balance of vital tissue, LIs were calculated and compared between stroke patients with and without hemiparesis and peers. Furthermore, the relationship between global CBF and age at examination, and sex was examined in stroke patients and peers.

### Cerebral perfusion balance in stroke patients and peers

In the hemispheres, the MCA territory, and the MCA anterior and middle subregions stroke patients showed an imbalanced perfusion to the contralesional hemisphere (positive LIs). In this respect, stroke patients differed significantly from peers, who had balanced LIs (values near 0). No effects were observed in the vascular territory of the ACA and the posterior part of the MCA. The majority of the cortical sensorimotor network is located in the MCA anterior and MCA middle subregions (primary motor cortex, supplementary motor cortex, premotor cortex, somatosensory cortex). It can therefore be assumed that perfusion balance in motor areas is directly related to motor outcome. Cortical areas of the ACA territory and the posterior part of the MCA are less strongly involved in movement, which also explains the absence of differences in the laterality indices in these areas between patients with and without hemiparesis and peers.

Wiest et al., who examined CBF lateralization in adults in the (sub)acute phase of AIS, showing successful and incomplete motor recovery, reported similar results [12]. Both Wiest et al. and the present study found that the patients with poor motor abilities (incomplete recovery, hemiparesis) showed a more imbalanced perfusion than patients with good motor abilities (successful recovery, no hemiparesis). In contrast to the successfully recovered group (Wiest et al.), who had balanced cerebral perfusion comparable to healthy controls, the patients without hemiparesis in the present study had significantly more imbalanced perfusion than peers.

These observations lead to the following conclusions. First, the findings in children with chronic AIS seem to be similar to those in adults in the (sub)acute phase of AIS. Second, as CBF was measured only in anatomically intact tissue, the LIs calculated in patients mirror perfusion asymmetries of non-ischemic regions. This suggests that, after AIS, perfusion balance is altered even in vital tissue. CBF of vital tissue is not altered on a global level (no difference in global CBF between patients and peers), but rather relatively (LIs). Third, the imbalanced LIs in patients without hemiparesis suggest that even where there is no motor impairment, cerebral perfusion might be altered after AIS. This concept is subsumed in hypothesis C) and is supported by the significant negative correlation between LIs and manual ability; the less imbalanced the LIs, the better the manual abilities. Again, being less involved in movement, the ACA territory showed no significant correlation with manual ability.

Not only perfusion balance, but also stroke volume influences motor function, as suggested by the correlation between stroke volume and ABILHAND-Kids measures in our study. Also, Wiest et al. observed that the incompletely recovered group had more extensive lesions than the successfully recovered group [12]. Differences in stroke volume, however, were not found between our patient groups.

Studying CBF LIs has some advantages over absolute CBF values. Despite the age-dependency of CBF [30–32,33] no age-adjusted calculations were needed to compare the groups with different age distributions, because LIs are relative measures. In addition, age-specific MRI masks are needed to define brain regions of interest during development. By using LIs, each participant served as an intraindividual reference, making normalization of ASL data in the MCA and MCA middle territory unnecessary.

### Relationship between global CBF and age and sex

Consistent with previous studies, global CBF decreased with increasing age throughout childhood and adolescence. However, studies differ in the observed course of age-related CBF changes [30–32]. In the present study, global CBF decreased linearly, whereas previous studies reported a non-linear decrease with global CBF being stable until late childhood, then decreasing before reaching adult levels [30–32]. Two studies reported that global CBF then increased slightly again [31,32]. Differences in sample size, age distribution in the samples, ASL protocols, and normalization or arousal during the scan might contribute to this divergence. As CBF mirrors the brain’s metabolic demands, the decline of global CBF with increasing age suggests that these demands also decrease with age. Indeed, cerebral glucose utilization was observed to be age-dependent and to show a similar pattern to global CBF. These age-related changes in glucose utilization and CBF may reflect the changing synaptic density during development of the brain [30,34]. Synaptic proliferation during the first years of life causes glucose utilization to increase and results in an excess of synapses. During subsequent synaptic pruning, inactive neuronal connections are eliminated and well-adjusted neuronal networks develop. The brain’s metabolic demands decrease, reflected by the decline in glucose utilization [34]. What remains unclear is why global CBF in female patients increased in the present study. Possible explanations include the small sample size (8 female patients) or alterations of global CBF due to AIS. Studies with larger sample sizes are desirable.

Concerning the relationship of global CBF with sex, a significant sex difference was found in peers. However, when adjusted for age at examination, this difference was no longer evident. Thus, global CBF seems to relate more strongly to age than to sex. Previous study findings vary: Biagi et al. reported no sex differences [30], Taki et al. observed regional CBF to be higher in females than in males [32], and Satterthwaite et al. found the following: At younger ages, regional CBF was lower in females than in males. Then, male CBF declined at a higher rate, resulting in lower CBF values in males than in females in mid-adolescence. In late adolescence, this difference was further accentuated by an increase in CBF in females [31]. The present study also found that CBF was initially higher in males but then decreased faster than CBF in females, resulting in higher CBF in females around 20 years. Satterthwaite et al. suggested that these sex differences could be an effect of rising estrogen levels during female puberty [31].

### Limitations

Our study was subject to the following limitations. First, the sample size is relatively small. However, the incidence of AIS is low and the sample sizes in comparable studies were even smaller [11]. Second, due to poor signal to noise ratio, accurately estimating CBF by the ASL method can be challenging: deviations up to 5% are likely (Andrea Federspiel, personal communication). Owing to physiological differences between children and adults, however, a previous study found the signal to noise ratio to be 70% higher than in adults [35]. Third, ASL is sensitive to vascular pathologies, such as stenoses, which delay the inflow of labeled blood to the slice of interest. As the blood relaxes with time, this may result in a smaller signal at the time of image acquisition and an underestimation of CBF [13]. In the present study no MR angiography was performed and the etiology of strokes varied greatly; hence no conclusion can be drawn about the relationship between vascular pathologies and CBF. However, since none of the included patients were treated by mechanical or intravenous thrombolysis, no influence of vascular treatment on CBF can be assumed. Fourth, earlier studies reported that age-related CBF changes vary between different brain regions [30,31,36]. To complement these findings, CBF could be analyzed in smaller, more specific brain regions in the future. Fifth, CBF was reported to show wide intersubject variability, even when corrected for age and sex [37]. To evaluate the development of CBF with increasing age, a longitudinal design might be more suitable than a cross-sectional one.

## Conclusions

Childhood stroke is rare but has a severe impact on a child’s life. Understanding the characteristics of cerebral perfusion in typically developing peers and stroke patients is important as it enables the understanding of healthy perfusion development and might help to predict and ameliorate stroke outcome. The present study shows that CBF decreases throughout healthy development. CBF in vital tissue is significantly less balanced in stroke patients than in peers. Patients with hemiparesis showed slightly more imbalanced perfusion (not statistically significant) than patients without hemiparesis, especially in motor areas. However, even in patients without hemiparesis, CBF is likely altered in the long-term. In future studies, the combination of resting state network characteristics or structural parameters of the brain with perfusion measures such as CBF can enable a better understanding of recovery mechanisms after stroke in childhood.

## Supporting information

S1 Table Detailed patient information. (XLSX)

S2 Table Absolute cerebral blood flow values. (PDF)
