# Supplementary material for: Cerebral blood flow imbalance is associated with motor outcome after pediatric arterial ischemic stroke
Source: PLoS One. 2019 Oct 11;14(10):e0223584. doi: 10.1371/journal.pone.0223584 (PMC6788710; doi:10.1371/journal.pone.0223584)
Supplement: S2 Table — (PDF) [file pone.0223584.s002.pdf]

SUPPLEMENTAL MATERIAL

Arterial Spin Labeling and Motor Outcome after Pediatric Arterial Ischemic Stroke

Rebekka Leistner, MMed[1]; Regula Everts, PhD[1,2]; Andrea Federspiel, PhD[3]; Salome Kornfeld, PhD[1,2]; Nedelina Slavova, MD[4]; Leonie Steiner, MSci[1]; Roland Wiest, MD[4]; Maja Steinlin, PhD[1,2]; Sebastian Grunt, MD, PhD[1]
1 Division of Neuropediatrics, Development and Rehabilitation, University Children's Hospital, Inselspital, Bern, Switzerland
2 Center for Cognition, Learning and Memory, University of Bern, Bern, Switzerland
3 Psychiatric Neuroimaging Unit, Translational Research Center, University Hospital of Psychiatry, University of Bern, Bern, Switzerland
4 Department of Diagnostic and Interventional Neuroradiology, Inselspital, Bern University Hospital, Bern, Switzerland

Address for Correspondence
Regula Everts, PhD
Division of Neuropediatrics, Development and Rehabilitation
University Children's Hospital, Inselspital
CH-3010 Bern
Switzerland
E-Mail: regula.everts@insel.ch
Phone: +41 31 632 94 24
Fax: +41 31 632 44 37

Supplemental Tables

Table I. Detailed patient data.

ID	Sex (m/f)	Age at stroke (y)	Age at examination (y)	Stroke side	Stroke location	Stroke Volume	PSOM (sensori-motor score)	Motor outcome	ABILHAND-Kids
2	f	4.00	6.08	left	cortical	0.0049	0	no hemiparesis	3.71
3	m	14.50	22.67	left	subcortical	3.7905	0	no hemiparesis	5.04
4	f	10.41	20.83	left	subcortical	0.0056	0.5	hemiparesis is right	6.68
5	f	11.75	15.50	right	subcortical	0.0063	0	no hemiparesis	6.68
7	m	6.25	18.42	left	combined cortical/subcortical	1.0792	1	hemiparesis is right	1.03
9	f	3.42	19.92	right	combined cortical/subcortical	0.3458	1	hemiparesis is left	4.38
11	f	7.83	15.33	left	subcortical	0.0227	0	no hemiparesis	6.68
12	m	3.50	11.42	left	subcortical	0.0076	0.5	hemiparesis is right	6.56
13	m	5.75	13.17	left	combined cortical/subcortical	3.4709	0	no hemiparesis	3.51
17	m	14.33	18.75	left	combined cortical/subcortical	0.0497	0	no hemiparesis	5.04
18	m	5.33	8.75	left	subcortical	7.7426	1	hemiparesis is right	1.07
19	m	1.50	9.42	left	subcortical	0.9983	0.5	hemiparesis is right	3.18

23	m	6.67	11.67	left	combined cortical/subcortical	0.0114	0	no hemiparesis	6.41
32	f	14.67	18.50	left	combined cortical/subcortical	0.0037	0	no hemiparesis	6.35
33	f	9.83	20.75	left	subcortical	11.6820	2	hemiparesis is right	2.12
35	m	0.00	9.50	left	cortical	0.0180	0	no hemiparesis	4.38
37	f	0.00	13.42	left	cortical	2.6464	1	hemiparesis is right	2.17
40	m	0.00	7.67	left	combined cortical/subcortical	1.0418	2	hemiparesis is right	0.34
54	m	0.00	10.75	left	cortical	0.0171	0	no hemiparesis	4.38
60	m	1.17	16.75	left	subcortical	0.0862	0	no hemiparesis	6.68

Stroke volume is given as [cm3]/total brain volume[cm3].
